# Beef Cattle Behavior Recognition Based on Nighttime Farm Videos via Spatio-Temporal Enhancement and Dynamic Fusion

**DOI:** 10.3390/ani16121881

**Published:** 2026-06-17

**Authors:** Yamin Han, Zhenyu Zhang, Wenchao Zhang, Shichao Cao, Yang Sun, Zixin Jia, Danyang Wu, Lyuwen Huang, Hongming Zhang

**Affiliations:** 1 College of Information Engineering, Northwest A&F University, Yangling 712100, China; yaminhan@nwafu.edu.cn (Y.H.); 2023051105@nwafu.edu.cn (Z.Z.); 2024056286@nwafu.edu.cn (W.Z.); caoshichao@nwafu.edu.cn (S.C.); sunyang@nwafu.edu.cn (Y.S.); 2023013349@nwafu.edu.cn (Z.J.); danyangwu.cs@gmail.com (D.W.); 2 Shaanxi Agricultural Information Intelligent Sensing and Analysis Engineering Technology Research Center, Yangling 712100, China

**Keywords:** beef cattle behavior recognition in the dark, spatio-temporal dark enhancement, dynamic fusion, joint training

## Abstract

Beef cattle behavior provides valuable information regarding their health status. Recently, deep convolutional network-based methods have achieved considerable results in beef cattle behavior recognition. However, their robustness under low-light or dark conditions remains limited, which restricts their application in real farm environments. To address this issue, this study constructed a realistic beef cattle behavior dataset in the dark, named Dark Beef Cattle Actions, which was collected under real nighttime farm conditions. The constructed dataset contains 1097 video clips collected from 30 beef cattle and covers 6 behavioral classes, including running, feeding, drinking, grooming, mounting, and fighting. Based on this dataset, we proposed a novel neural network architecture based on spatio-temporal dark enhancement and dynamic fusion for beef cattle behavior recognition in the dark. First, a spatio-temporal dark enhancement module was designed to improve dark video quality while preserving motion features. Second, a dynamic fusion module was introduced to adaptively fuse features from different branches and obtain more discriminative representations. In addition, a joint loss was adopted to optimize both dark enhancement and action recognition. Experimental results on the constructed dataset show that the proposed method achieved a weighted-averaged precision score of 88.47%, a weighted-averaged recall score of 80.18%, an accuracy score of 83.80%, and a weighted-averaged F1-score of 84.12%. Compared with other state-of-the-art methods, the proposed method achieved competitive performance in the recognition of night-time beef cattle behavior. These findings would provide support for intelligent livestock behavior recognition and monitoring in precision farming.

## Simple Summary

Precision livestock farming is essential for improving animal welfare, farm management, and production efficiency. Recently, automatic beef cattle behavior recognition has become a promising solution for continuous and intelligent livestock monitoring. However, nighttime farm videos are often affected by poor illumination, blurred behavioral regions, and weak motion information, making accurate behavior recognition difficult. In this study, we explore a novel nighttime beef cattle behavior recognition method based on spatio-temporal enhancement and dynamic feature fusion. The results have shown that our proposed method has the characteristics of accurate, robust, and illumination-adaptive recognition, which provides a potential solution for automatic beef cattle behavior monitoring in actual nighttime farm environments.

## 1. Introduction

The task of recognizing beef cattle behaviors has received considerable attention in both academia and industry due to its great significance for disease prevention, reproductive management, welfare assessment, and production efficiency in beef cattle farming [1,2,3,4]. Although cattle behavior recognition in videos recorded under normal illumination has achieved great success, the recognition of cattle behaviors in videos shot under low illumination remains an under-explored area with significant challenges. In practical farm environments, nighttime behaviors are closely associated with physiological needs, health status, comfort, social interactions, reproductive activities, and potential abnormal events. The lack of research on beef cattle behavior recognition in dark videos may lead to the neglect of certain behaviors that occur frequently at night, such as estrus, thereby causing economic losses. Therefore, there is a need for an efficient method to monitor beef cattle behaviors in the dark.

With the development of computer vision technologies, non-contact methods [5,6,7,8,9,10,11,12,13] have become mainstream and have achieved state-of-the-art performance due to their advantages of lower cost, reduced stress on animals, and higher efficiency. For example, Myat Noe et al. [5] compared multiple deep learning algorithms for automated black cattle detection and tracking, demonstrating the potential of visual perception techniques for continuous cattle monitoring. Previous studies [6,7,8,9] generally utilized 2D convolutional neural networks to extract spatial features from single-frame images or detected cattle image regions for beef cattle behavior recognition. In particular, Li et al. [8] improved YOLOv8n by introducing dynamic snake convolution and the BiFormer attention mechanism for multi-behavior recognition in group-housed beef cattle. Giannone et al. [9] fine-tuned an improved YOLOv8n model for individual dairy cow identification at feeding lanes and used the detection results for feeding behavior analysis. However, the lack of sufficient temporal information limits their ability to represent continuous behavioral dynamics. Other studies [10,11,12,13] employed pose estimation networks to obtain cattle keypoints or skeletal representations, which are subsequently used for behavior recognition. For example, Hua et al. [13] adopted an improved YOLOX-Pose to extract skeletal keypoints of dairy cows and then used PoseC3D to model the temporal dynamics of the skeleton sequence for behavior recognition. Unfortunately, these methods mainly depend on the quality of skeleton extraction, and their performance may degrade when keypoints are inaccurately estimated under occlusion or complex farm scenes.

To address these limitations, recent research [14,15,16,17,18] has increasingly focused on leveraging human action recognition methods [19,20,21,22,23] for animal behavior recognition tasks. These methods perform joint representation of spatial appearance information and temporal dynamics via techniques such as 3D CNN [14], CNN-LSTM [15,16], and spatio-temporal feature fusion [17,18]. For example, Wang et al. [14] proposed an efficient 3D CNN named E3D. It directly captured spatio-temporal information from video clips and improved recognition performance with fewer parameters and lower computational complexity. Yin et al. [15] proposed an EfficientNet-LSTM framework for dairy cattle behavior recognition. In this method, the spatial features extracted by EfficientNet were further fed into an LSTM for behavior classification. Wu et al. [16] adopted a CNN-LSTM architecture for basic behavior detection in individual dairy cattle. The results demonstrated the effectiveness of combining convolutional feature extraction with temporal dependency analysis. Fuentes et al. [17] achieved cattle behavior recognition by integrating frame-level appearance features with spatio-temporal contextual information. Tian et al. [18] further introduced the spatio-temporal feature fusion method Cattle-ES3D to enhance behavior representation across different spatial and temporal scales. However, those methods only focused on the behaviors of beef cattle in videos recorded under normal illumination, while ignoring the behaviors in dark videos that also contain valuable information.

Some scholars have focused on animal behavior recognition in the dark. Wu et al. [16] and Han et al. [24] evaluated the robustness of their proposed methods for behavior recognition under different illumination conditions. However, these studies have not developed specialized modules for low-light environments, resulting in poor recognition performance. Xiao et al. [25] improved duck flock behavior recognition across different illumination conditions by incorporating dual detection heads, channel attention, and an optimized loss function into the detection framework. Dai et al. [26] improved nighttime cow mounting detection by combining an illumination-adaptive Transformer with an efficient feature fusion detector. It remained effective under low-light conditions and severe target scale variations. Nevertheless, these methods [25,26] are primarily built upon image-based detection paradigms. Such designs neglect motion dependency, thereby limiting their ability to learn effective spatio-temporal representations for complex behavior recognition.

Focusing on those limitations, in this work, we first collected a realistic beef cattle behavior dataset in the dark, named Dark Beef Cattle Actions, which was recorded under real nighttime farm conditions. It contained behaviors such as feeding, drinking, grooming, mounting, fighting, and running. Based on the collected dataset, we proposed a novel neural network based on spatio-temporal enhancement and dynamic fusion for beef cattle behavior recognition in the dark. We first developed a spatio-temporal enhancement module to improve dark video quality while preserving spatial and temporal details. Then, multi-stream combining original dark, spatio-temporal-enhanced, and histogram-equalized inputs are fed into a shared CNN feature extractor to obtain complementary features. To further improve the recognition accuracy, a dynamic fusion module adaptively fuses features from multiple streams by balancing local and global contextual information. Besides, the joint training loss function is utilized to further optimize both dark enhancement and action recognition. The proposed method achieved a precision score of 88.47%, a recall score of 80.18%, an accuracy score of 83.80%, and an F1-score of 84.12%.

The main contributions of our work can be summarized as follows: (1) we propose a novel neural network architecture based on spatio-temporal dark enhancement and dynamic fusion for beef cattle behavior recognition in the dark; (2) a spatio-temporal enhancement module is designed to improve video quality in dark scenes while preserving behavior-relevant semantic information. To effectively exploit complementary information, a novel dynamic fusion strategy is proposed to adaptively fuse features from multiple inputs; (3) the Dark Beef Cattle Actions dataset collected under real nighttime farm conditions is introduced for open benchmark verification. Experimental results on the constructed dataset showed that the proposed method achieved the most competitive performance for beef cattle behavior recognition in the dark.

## 2. Materials and Methods

### 2.1. Dataset

**Figure 1:**
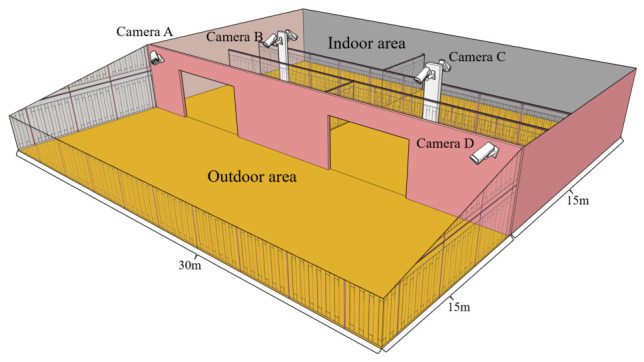
Schematic diagram of the video recording setup.

To recognize beef cattle behaviors in dark environments, we first constructed a dataset containing real nighttime beef cattle behaviors, named Dark Beef Cattle Actions. The experimental subjects were 30 female Angus cattle, all approximately 22 months of age. The videos were collected in the indoor area of a beef cattle farm at Shaanxi KingBull Beef Industry in Yangling District, Xianyang, Shaanxi, China. Each cattle pen was approximately 30 m in length and 15 m in width. One Hikvision network surveillance camera was installed in each pen on a supporting pillar of the barn at a height of approximately 3.5 m. The camera was angled downward to cover the entire activity area of the cattle. The videos were recorded at a resolution of 1920 × 1080 pixels with a frame rate of 25 fps. During nighttime data acquisition, each pen was monitored by an independent surveillance device without the use of multiple synchronized cameras. All cameras operated under standard farm surveillance conditions using infrared night-vision illumination, without additional visible-light supplementation. Technical details of the beef cattle data acquisition are shown in Figure 1.

The videos were collected from November 2023 to March 2024. In our study, the video sampling time ranged from 19:00 to 07:00 of the following day. The constructed dataset, referred to as Dark Beef Cattle Actions, contains 1097 video clips. Each clip ranges from 50 to 400 frames in length. Based on manual annotation by animal experts, these clips were labeled into six behavioral categories: running, feeding, drinking, grooming, mounting, and fighting. The definitions of all behavioral categories were established with reference to previously published cattle behavior classification standards [27,28]. The annotation process was conducted by three annotators. Before formal annotation, all annotators received guidance from professionals with academic backgrounds in animal science to ensure a consistent understanding of beef cattle behaviors. A unified annotation criterion was established before labeling, and all video clips were independently checked according to the definitions of the six behavioral categories. Inter-observer agreement was assessed by comparing the labels assigned by the three annotators, and inconsistent labels were further reviewed by professionals to determine the final annotations.

**Figure 2:**
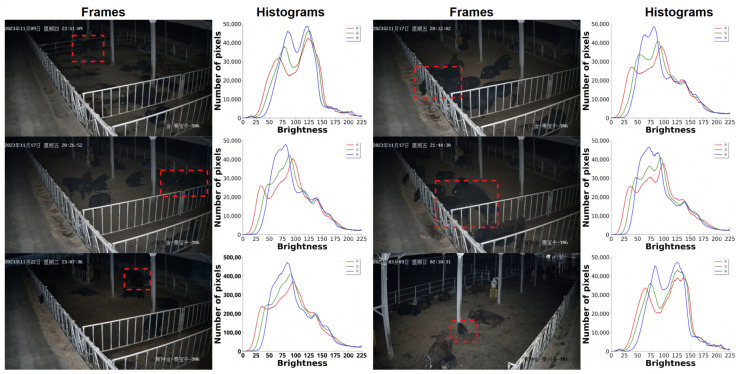
Sampled video frames of different beef cattle behaviors under low-light conditions and their corresponding RGB histograms. The Frames column shows the sampled video frames, while the Histograms column presents the RGB color histograms of each frame. For better visualization, the behavioral regions are marked with red dashed boxes.

Figure 2 displays sample video frames with action labels. In Figure 2, RGB histograms are used to illustrate the differences between video sequences of different behavioral categories. Additionally, Table 1 provides a detailed description of each behavior.

**Figure 3:**
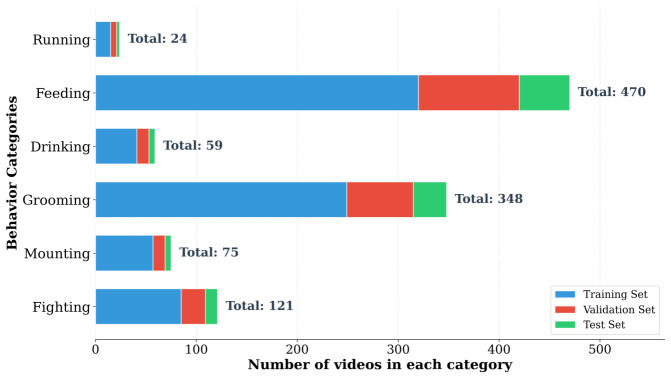
Overview of the Dark Beef Cattle Actions dataset.

To evaluate the behavior recognition performance of the proposed model, the 1097 video clips were divided into training, validation, and test sets at an approximate ratio of 7:2:1, consisting of 767, 220, and 110 clips, respectively. To reduce potential information leakage between temporally adjacent clips, the dataset was split chronologically into training, validation, and test sets according to video acquisition time. Specifically, clips from the earlier period were used for model training, clips from the middle period were used for validation, and clips from the later period were used for testing. The statistical results are shown in Figure 3. Specifically, the running category contains 14 training clips, 6 validation clips, and 4 test clips. The feeding category contains 329 training clips, 96 validation clips, and 45 test clips. The drinking category contains 41 training clips, 12 validation clips, and 6 test clips. The grooming category contains 246 training clips, 67 validation clips, and 35 test clips. The mounting category contains 52 training clips, 15 validation clips, and 8 test clips. The fighting category contains 85 training clips, 24 validation clips, and 12 test clips. It is noteworthy that behaviors in the dark, such as running, occur only rarely in real farming scenarios. Consequently, the dataset suffers from class imbalance, which poses additional challenges for model training and evaluation.

### 2.2. A Novel Network Based on Spatio-Temporal Enhancement and Dynamic Fusion (STED-Net)

#### 2.2.1. Overview

**Figure 4:**
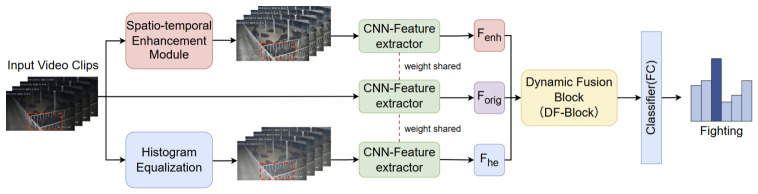
Architecture of the proposed STED-Net method. The red dashed boxes indicate the main regions where cattle behaviors occur.

Figure 4 shows the structure of the proposed STED-Net method. It mainly consists of two core components: the Spatio-Temporal Enhancement Module (STE-Module) and the Dynamic Fusion Block (DF-Block). Given an input video V, we construct a video clip $X^{\left(o\right)}=\left\{I^{\left(t_{1}\right)},I^{\left(t_{2}\right)},\ldots ,I^{\left(t_{N}\right)}\right\}$ consisting of *N* representative frames by uniform sampling [29]. Specifically, each clip is divided into *N* temporal segments, and one frame is sampled per segment (randomly during training, center frame during inference) to ensure consistent temporal coverage across clips of varying lengths. Subsequently, the sampled frames $X^{\left(o\right)}$ are fed into two parallel processing branches. The upper branch inputs $X^{\left(o\right)}$ into the proposed STE-Module to improve illumination quality, denoted as $X^{\left(e\right)}=E\left(X^{\left(o\right)}\right)$, where E(·) denotes the nonlinear mapping. The lower branch applies histogram equalization to the $X^{\left(o\right)}$ to improve image contrast details in dark regions, which is denoted as $X^{\left(h\right)}=H\left(X^{\left(o\right)}\right)$, where H(·) denotes the histogram equalization operation. Afterward, a convolutional neural network with shared weights is employed to extract feature representations from the original video clip $X^{\left(o\right)}$ and the two enhanced clips, $X^{\left(e\right)}$ and $X^{\left(h\right)}$. The resulting features $F^{\left(o\right)}$, $F^{\left(e\right)}$, and $F^{\left(h\right)}$ are subsequently integrated through the proposed DF-Block to obtain adaptively fused feature representations. Finally, the fused features are fed into a temporal average pooling layer and a fully connected layer for the final video classification result.

#### 2.2.2. Spatio-Temporal Enhancement Module (STE-Module)

**Figure 5:**
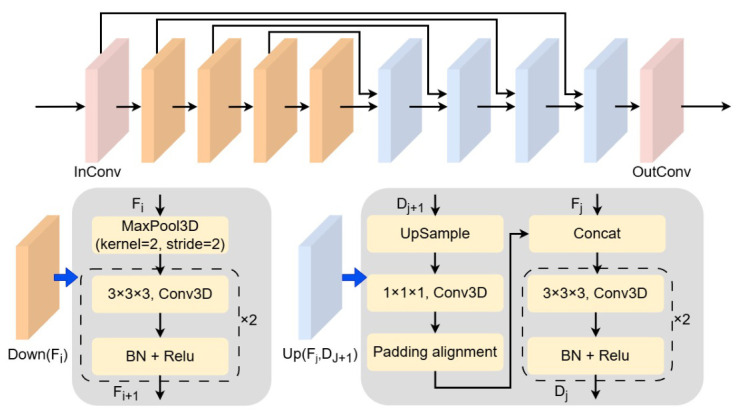
Architecture of the proposed STE-Module. InConv and OutConv denote the initial and the final convolution layer, respectively.

Unlike the method proposed by Tran et al. [30] that perform low-light brightening as an independent preprocessing operation, the proposed STE-module is embedded into the recognition network and jointly optimized with the classification task, enabling it to enhance behavior-related spatio-temporal features rather than only improving visual quality. Figure 5 illustrates the overall architecture of the proposed STE-module. The network follows a hierarchical downsampling–upsampling design, where the former is used for feature extraction and the latter for feature reconstruction.

Based on the sampled video clips $X^{\left(o\right)}=\left\{I^{\left(t_{1}\right)},I^{\left(t_{2}\right)},\ldots ,I^{\left(t_{N}\right)}\right\}$ mentioned above, the proposed STE-module is employed to improve the brightness of the video clips. The input clip $X^{\left(o\right)}$ is first fed into an initial convolution layer to obtain shallow feature representations, denoted as: $F_{1}=\mathrm{InConv}\left(X^{\left(o\right)}\right)$, where InConv(·) denotes the input convolution block composed of two consecutive 3D convolution operations and nonlinear activation functions. The resulting features $F_{1}$ are then fed into a series of ${\mathrm{Down}}_{i}$ layer, which consists of 3D convolution and max-pooling operations. The generated multi-scale spatio-temporal features $F_{i+1}$ are defined as:

(1) $$F_{i+1}={\mathrm{Down}}_{i}\left(F_{i}\right),\;i=1,2,3,4$$

where $F_{i}$ represents the input spatio-temporal feature map to the *i*-th layer, and the *i*-th layer is defined as:

(2) $${\mathrm{Down}}_{i}\left(F_{i}\right)=\mathrm{DoubleConv}\left({\mathrm{Pool}}_{i}\left(F_{i}\right)\right)$$

where ${\mathrm{Pool}}_{i}\left(\cdot \right)$ denotes the 3D max-pooling operation and DoubleConv(·) denotes two consecutive 3D convolutions with nonlinear activations. As the ${\mathrm{Down}}_{i}$ layer goes deeper, the feature maps $F_{i}$ gradually decrease in temporal-spatial resolution while increasing in semantic abstraction. They provide spatio-temporal representations at different scales. These features progressively capture richer contextual information and serve as the basis for subsequent reconstruction.

Afterwards, the network gradually restores the feature-map resolution through upsampling and integrates skip-connected features from the corresponding ${\mathrm{Down}}_{i}$ layers to compensate for the detailed information that may be lost during feature extraction. Considering that feature maps from different layers may have inconsistent sizes, a padding alignment operation is introduced during feature reconstruction to ensure dimensional consistency before feature concatenation. The feature reconstruction process can be defined as:

(3) $$D_{j}={\mathrm{Up}}_{j}\left(D_{j+1},F_{j}\right),\;i=1,2,3,4$$

where $D_{j+1}$ denotes the input feature from the previous feature reconstruction stage, $F_{j}$ denotes the skip-connected feature at the corresponding scale from ${\mathrm{Down}}_{j-1}$. For the initial feature reconstruction stage, the feature is directly defined as $D_{5}=F_{5}$. And the *j*-th layer is defined as:

(4) $${\mathrm{Up}}_{j}\left(D_{j+1},F_{j}\right)=\mathrm{DoubleConv}\left(\mathrm{Concat}({\mathrm{UpSample}}_{j}\left(D_{j+1}\right),F_{j})\right)$$

where ${\mathrm{UpSample}}_{j}\left(\cdot \right)$ denotes the up-sampling operation, and Concat(·,·) denotes channel-wise concatenation. The reconstructed features $D_{i}$ progressively restore the temporal-spatial resolution of the feature maps.

Finally, the enhanced video clips $X^{\left(e\right)}$ is reconstructed through the output convolution:

(5) $$X^{\left(e\right)}=\mathrm{OutConv}\left(D_{1}\right)$$

Through the above formulations, the STE-Module can enhance low-light video representations by modeling spatial–temporal dependencies, preserving temporal consistency, and recovering structural details.

#### 2.2.3. Dynamic Fusion Block (DF-Block)

**Figure 6:**
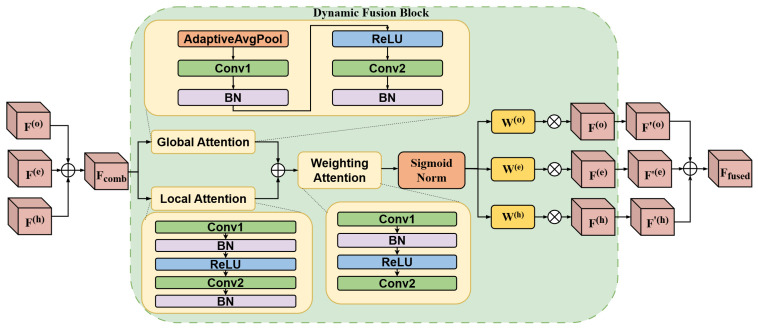
The proposed DF-Block architecture.

Unlike the existing attention feature fusion method proposed by Dai et al. [31], which mainly focuses on attentional fusion of general feature maps through local and global attention, the proposed DF-Block further introduces branch-level dynamic weighting to explicitly adjust the contributions of different feature streams during fusion. This allows the DF-Block to adaptively balance complementary information from different enhancement streams while suppressing redundant or noisy features caused by poor illumination. Figure 6 shows the overall architecture of the DF-Block employed.

Based on the three video frame clips $\left[X^{\left(o\right)},X^{\left(e\right)},X^{\left(h\right)}\right]$, the shared CNN-based feature extraction is further performed to extract the corresponding feature representations $F^{\left(o\right)}$, $F^{\left(e\right)}$, and $F^{\left(h\right)}$, respectively. The three feature maps have the same dimensionality, which ensures that they can be directly used for subsequent dynamic fusion.

First, these three features are initially combined by element-wise addition to generate the combined feature $F_{\mathrm{comb}}=\sum_{i\in \{o,e,h\}}F^{\left(i\right)}.$ Subsequently, $F_{\mathrm{comb}}$ is processed by two parallel branches to extract global and local contextual information, respectively. The global attention branch, denoted as $F_{\mathrm{comb}}^{\left(G\right)}$, captures global contextual representations over the entire receptive field. Specifically, the spatial dimension of $F_{\mathrm{comb}}$ is first compressed via the adaptive average pooling operation AdaptiveAvgPool(·) to aggregate macroscopic feature information. And the resulting feature is then transformed through nonlinear mapping to obtain global feature representations. The calculation formula for the global attention branch can be expanded as follows:

(6) $$F_{\mathrm{comb}}^{\left(G\right)}={\mathrm{Conv}}_{2}\left({\mathrm{Conv}}_{1}\left(\mathrm{AdaptiveAvgPool}\left(F_{\mathrm{comb}}\right)\right)\right)$$

where ${\mathrm{Conv}}_{1}$ and ${\mathrm{Conv}}_{2}$ denote two convolutional layers followed by activation functions.

The Local Attention branch, denoted as $F_{\mathrm{comb}}^{\left(L\right)}$, focuses on extracting fine-grained local visual features at the original spatial resolution. It does not compress the spatial dimension but directly uses convolution operations to capture high-frequency local details, compensating for the loss of detail caused by global operations. The calculation formula for this branch can be expanded as:

(7) $$F_{\mathrm{comb}}^{\left(L\right)}={\mathrm{Conv}}_{2}\left({\mathrm{Conv}}_{1}\left(F_{\mathrm{comb}}\right)\right)$$

The outputs of $F_{\mathrm{comb}}^{\left(G\right)}$ and $F_{\mathrm{comb}}^{\left(L\right)}$ are then combined by element-wise addition and fed into the Weighting Attention module, which is used to generate the adaptive weights $W_{i}$ for each input branch. The Weighting Attention module also consists of ${\mathrm{Conv}}_{1}$ and ${\mathrm{Conv}}_{2}$, each comprising a convolutional layer followed by an activation function, where the number of output channels corresponds to the number of branches in the input feature. The generated values are further mapped by the sigmoid function and the normalization operation into the final branch-wise weights:

(8) $$\begin{array}{c} W=\mathrm{Norm}\left({\mathrm{Conv}}_{2}\left({\mathrm{Conv}}_{1}\left(F_{\mathrm{comb}}^{\left(G\right)}+F_{\mathrm{comb}}^{\left(L\right)}\right)\right)\right) \end{array}$$

After obtaining the adaptive weights $W={\left[W^{\left(o\right)},W^{\left(e\right)},W^{\left(h\right)}\right]}^{⊤}$,the features from the three branches are reweighted as:

(9) $$\left[F^{\prime (o)},F^{\prime (e)},F^{\prime (h)}\right]=W⊙\left[F^{\left(o\right)},F^{\left(e\right)},F^{\left(h\right)}\right]$$

where $F^{\prime (o)}$, $F^{\prime (e)}$, and $F^{\prime (h)}$ represent the weighted features of the original branch, the enhancement branch, and the histogram-equalization branch, respectively. The final fused feature $F_{\mathrm{fused}}$ is then obtained by summing these weighted features, which can be formulated as follows:

(10) $$F_{\mathrm{fused}}=\sum_{i\in \{o,e,h\}}F^{\prime (i)}$$

Afterwards, the fused feature $F_{\mathrm{fused}}$ is fed into the classification module that consists of a temporal average pooling and a fully connected layer to obtain the final behavior classification result of the input video.

#### 2.2.4. Joint Optimization Loss for Enhancement and Recognition

To enable the proposed framework to simultaneously improve low-light video quality and behavior recognition performance, a joint optimization strategy is adopted during training. Specifically, the overall objective function is designed from two complementary perspectives: enhancement-oriented supervision and classification-oriented supervision. The former constrains the enhanced video clips in terms of brightness improvement, temporal consistency, structural fidelity, and perceptual quality, while the latter guides the network to learn discriminative representations for downstream behavior classification. By jointly optimizing these objectives in a unified end-to-end manner, the proposed model is encouraged not only to generate temporally stable and visually enhanced video clips, but also to preserve behavior-relevant semantic cues that are critical for accurate recognition under low-light conditions.

To avoid an overly lengthy and less interpretable formulation caused by directly summing all loss terms into a single objective, the overall loss $L_{total}$ is first decomposed into a classification loss $L_{cls}$ and an enhancement loss $L_{enh}$. Specifically, the total loss function is defined as:

(11) $$L_{total}=L_{cls}+\lambda_{enh}L_{enh}$$

where $L_{cls}$ denotes the classification loss for behavior recognition, and $\lambda_{enh}$ denotes the composite loss for low-light video enhancement. The classification loss $L_{cls}$ is implemented as the cross-entropy loss, which is used to supervise behavior category prediction. It is defined as:

(12) $$L_{cls}=-\sum_{c=1}^{C}y_{c}\log\left({\hat{y}}_{c}\right)$$

where *C* denotes the number of behavior categories, $y_{c}$ denotes the ground-truth label of the *c*-th class, and ${\hat{y}}_{c}$ denotes the predicted probability for the corresponding class.Meanwhile, the enhancement loss $L_{enh}$ is introduced to constrain the low-light video enhancement process and improve the visual quality of the enhanced video, which is formulated as:

(13) $$L_{enh}=L_{DCE}+\lambda_{stc}L_{stc}$$

where $L_{DCE}$ denotes the low-light enhancement loss inherited from Zero-DCE [32], and $L_{stc}$ denotes the spatio-temporal consistency loss introduced to preserve structural fidelity and temporal coherence in enhanced video sequences.

In this work, $L_{DCE}$ regularizes the enhancement process in terms of exposure, color balance, and smoothness, thereby enabling the network to generate visually natural and properly illuminated results, which is defined as:

(14) $$L_{enh}=L_{spa}+L_{exp}+W_{col}L_{col}+W_{tv_{A}}L_{tv_{A}}$$

where $L_{spa}$ enforces spatial consistency between the input and enhanced images, $L_{exp}$ constrains the local exposure level, $L_{col}$ promotes color constancy among different channels, and $L_{tv_{A}}$ regularizes the smoothness of the estimated enhancement curves. The formulations and parameter settings of these loss terms are kept consistent with those reported in Zero-DCE [32].

The adopted Zero-DCE loss effectively improves frame-level illumination quality. However, it does not explicitly constrain temporal coherence across video frames. As a result, the enhancement model may generate visually improved individual frames but still fail to maintain stable inter-frame consistency. Considering that temporal stability is crucial for downstream behavior recognition, the spatio-temporal consistency loss $L_{stc}$ is introduced to enforce coherent luminance variation, structural continuity, and perceptual consistency across consecutive frames. Specifically, the introduced spatio-temporal consistency constraints consist of the temporal consistency loss $L_{tc}$, the 3D structural similarity loss $L_{3DSSIM}$, and the perceptual loss $L_{per}$. These loss terms are designed to jointly maintain temporal coherence, structural continuity, and perceptual consistency in the enhanced video sequence. The overall spatio-temporal consistency loss $L_{stc}$ integrates the above three components into a unified formulation:

(15) $$L_{stc}=\lambda_{tc}L_{tc}+\lambda_{ssim}L_{3DSSIM}+\lambda_{per}L_{per}$$

where $\lambda_{tc}$, $\lambda_{ssim}$, and $\lambda_{per}$ are weighting coefficients that control the relative contributions of each loss.

The temporal consistency loss is introduced to suppress frame-to-frame luminance fluctuation and preserve smooth temporal transitions in the enhanced video sequence. It is formulated as:

(16) $$L_{tc}=\frac{1}{T-1}\sum_{t=1}^{T-1}∥\left(X_{t+1}^{\left(e\right)}-X_{t}^{\left(e\right)}\right)-\left(X_{t+1}^{\left(o\right)}-X_{t}^{\left(o\right)}\right){∥}_{1}$$

where $X_{t}^{\left(e\right)}$ and $X_{t+1}^{\left(e\right)}$ denote the enhanced frames at time steps *t* and t+1, respectively, while $X_{t}^{\left(o\right)}$ and $X_{t+1}^{\left(o\right)}$ represent the corresponding reference frames. *T* is the total number of frames in the video clip, and ${∥\cdot ∥}_{1}$ denotes the $\ell_{1}$ norm. By minimizing the difference between temporal intensity variations in the enhanced sequence and those in the reference sequence, this loss encourages the model to maintain consistent inter-frame changes and reduces temporal flickering artifacts.

To further preserve local structural continuity in both spatial and temporal domains, the 3D structural similarity loss is adopted following the 3D-SSIM measure proposed by Zeng and Wang [33], which is defined as:

(17) $$L_{3DSSIM}=1-\frac{1}{N}\sum_{w=1}^{N}{\mathrm{SSIM}}_{3D}\left(X^{\left(e\right)},X^{\left(o\right)}\right)$$

where *N* is the total number of sampled local 3D patches.

To enhance the perceptual fidelity of the restored video frames, a VGG-based perceptual loss is further adopted. Specifically, we employ a VGG-16 network pretrained on ImageNet as the fixed feature extractor, and the feature maps extracted from the selected convolutional layers are used to measure the perceptual discrepancy between the enhanced and reference frames. In this study, four intermediate feature layers are selected for perceptual loss computation, corresponding to indices 3, 8, 15, and 22 in the feature extraction module of the VGG-16 network. The parameters of the pretrained VGG network are frozen during training.

Before being fed into the VGG network, both the enhanced frames and the reference frames are normalized using the standard ImageNet preprocessing strategy. The perceptual loss is formulated as:

(18) $$L_{per}=\frac{1}{L}\sum_{l=1}^{L}{∥\phi_{l}\left(X^{\left(e\right)}\right)-\phi_{l}\left(X^{\left(o\right)}\right)∥}_{2}^{2}$$

where $\phi_{l}\left(\cdot \right)$ denotes the feature map extracted from the *l*-th selected layer of a pretrained feature extractor, *L* is the number of selected layers, and ${∥\cdot ∥}_{2}^{2}$ represents the squared $\ell_{2}$ norm. $X^{\left(e\right)}$ and $X^{\left(o\right)}$ denote the enhanced and reference inputs, respectively. By minimizing the discrepancy between deep feature representations, this loss encourages the enhanced results to remain consistent with the reference sequence in terms of high-level semantic structure and visual perception.

In summary, the overall loss function $L_{total}$ in this study integrates enhancement supervision $L_{DCE}$, spatio-temporal consistency constraints $L_{stc}$, and behavior classification supervision $L_{cls}$ into a unified optimization framework. The enhancement loss improves the illumination and visual quality of low-light video frames, the spatio-temporal consistency losses suppress temporal instability and preserve structural as well as perceptual continuity across consecutive frames, and the classification loss further promotes the learning of behavior-discriminative features. In our experiments, these above weights were set to $\lambda_{enh}=0.05$, $\lambda_{stc}=2$, $W_{col}=0.5$, $W_{tv_{A}}=20$, $\lambda_{tc}=2.0$, $\lambda_{ssim}=1.5$, and $\lambda_{per}=0.5$. These values were empirically determined according to the validation performance and kept fixed in all experiments. Benefiting from the joint optimization of these complementary objectives, the proposed framework achieves a better balance between video enhancement quality and downstream behavior recognition performance in low-light scenarios.

### 2.3. Evaluation Indicators

In this work, the evaluation of behavior recognition performance is conducted using four key metrics: Precision, Recall, F1-score, and Accuracy. Precision measures the proportion of correctly identified positive samples among all predicted positives, while Recall quantifies the proportion of correctly detected positives out of all actual positive instances. The F1-score provides a balanced assessment by combining Precision and Recall into a single harmonic mean, reflecting the trade-off between these two metrics. Accuracy represents the overall correctness of classification, defined as the ratio of correctly predicted samples to the total number of samples. The computation of these metrics follows Equations (19)–(22).

(19) $$\mathrm{Precision}=\frac{TP}{TP+FP}$$

(20) $$\mathrm{Recall}=\frac{TP}{TP+FN}$$

(21) $$F1\mathrm{-score}=\frac{2\times \mathrm{Precision}\times \mathrm{Recall}}{\mathrm{Precision}+\mathrm{Recall}}$$

(22) $$\mathrm{Accuracy}=\frac{TP+TN}{TP+TN+FP+FN}$$

where TP, FP, FN, and TN are the numbers of true positives, false positives, false negatives, and true negatives, respectively. These metrics together provide a comprehensive evaluation of the model’s classification ability from different perspectives, ensuring a balanced assessment of precision, completeness, and overall reliability.

### 2.4. Experimental Setup

In this study, the proposed STED-Net model and all comparison methods were implemented using Python 3.8.0 and PyTorch 1.8.1. The experiments were conducted on a deep learning server running Ubuntu 20.04.3 LTS (Canonical Ltd., London, UK), equipped with an Intel Xeon Silver 4316 processor, 64 GB of memory, and two NVIDIA GeForce RTX 4090-24G GPUs, with CUDA 11.1 serving as the parallel processing environment. A 4 TB external hard drive was used for data storage and subsequent processing.

For the proposed STED-Net network, we adopted backbone weights pretrained on ARID [34]. During the training phase, we employed multiple flipping augmentation strategies (horizontal flipping, vertical flipping, and mirror flipping) to increase data diversity. Each input video segment consisted of 32 consecutive frames, and the image resolution was set to 256 × 256 × 3. The dropout rate was set to 0.8. The model was optimized using the AdamW optimizer with an initial learning rate of $1\times 10^{-5}$, a mini-batch size of 8, and a weight decay of $1\times 10^{-3}$. A ReduceLROnPlateau learning-rate scheduler was used with a patience value of 5. The best model was selected according to the highest validation Top-1 accuracy during training. It is worth noting that no additional class balancing strategy, class-weighted loss, or re-sampling strategy was adopted during training, and all behavior categories were trained under the original data distribution.

## 3. Results and Discussion

### 3.1. Comparisons with the State-of-the-Art Behavior Recognition Methods

Unlike previous studies that mainly focus on cattle action recognition under daylight conditions, the proposed STED-Net is designed for multi-class behavior recognition of group-housed cattle under low-light conditions. In addition, we compare the proposed STED-Net with a series of representative methods widely used in human action recognition, including C3D [35], TSN [29], I3D [36], R(2 + 1)D [37], TRN [38], SlowFast [39], TSM [40], TAM [41], TPN [42], and Swin-Transformer [43]. To ensure a fair comparison, all compared were re-trained and evaluated using identical experimental settings, including data splits, data augmentation strategy, pretraining setting, and number of training epochs. As shown in Table 2, the proposed STED-Net achieves 88.47% precision, 80.18% recall, 83.80% accuracy, and 84.12% F1-score, outperforming all competing methods across all evaluation metrics. Specifically, compared with the best-performing TSM, STED-Net improves precision by 1.72%, recall by 10.95%, accuracy by 17.84%, and F1-score by 7.11%.

In addition to recognition performance, computational efficiency is also analyzed to assess the practical feasibility of the proposed framework. Table 2 summarizes the model parameters and inference times of different behavior recognition methods on the test dataset, where inference time is measured in milliseconds (ms). Without any code-level optimization, STED-Net exhibits an inference time of 292.26 ms per sample. This increased computational cost mainly arises from the integrated video enhancement module. Therefore, although STED-Net improves recognition robustness under severe low-light conditions, its current computational cost may limit direct deployment on resource-constrained edge devices or strict real-time monitoring systems. In future work, more lightweight enhancement modules and branch designs will be explored to further reduce model size and inference latency.

**Figure 7:**
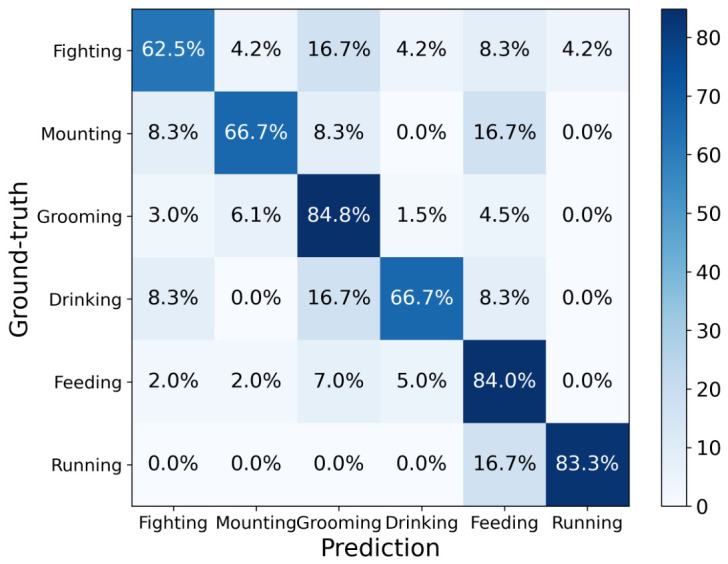
Confusion matrix of behavior recognition using the STED-Net method.

The confusion matrix of the proposed STED-Net method for six beef cattle behaviors is illustrated in Figure 7. Overall, the STED-Net method achieved recognition accuracies of 62.5%, 66.7%, 84.8%, 66.7%, 84.0%, and 83.3% for fighting, mounting, grooming, drinking, feeding, and running behaviors, respectively. These results demonstrate that the proposed STED-Net method is capable of accurately recognizing beef cattle behaviors in nighttime environments.

As shown in Figure 7, misclassifications mainly occur in fighting, mounting and drinking. Fighting and drinking are both partly misclassified as grooming, possibly because head contact, head-lowering postures, and local body movements become blurred under nighttime low-light conditions. For mounting, the main misclassification occurs when it is predicted as Feeding, accounting for 16.7% of the Mounting samples. This may be because mounting usually involves short-duration body contact and partial overlap between cattle. Overall, the misclassifications are mainly caused by low illumination, motion blur, occlusion, long camera distance, and the similarity of postures and interaction patterns among different behavior categories.

**Figure 8:**
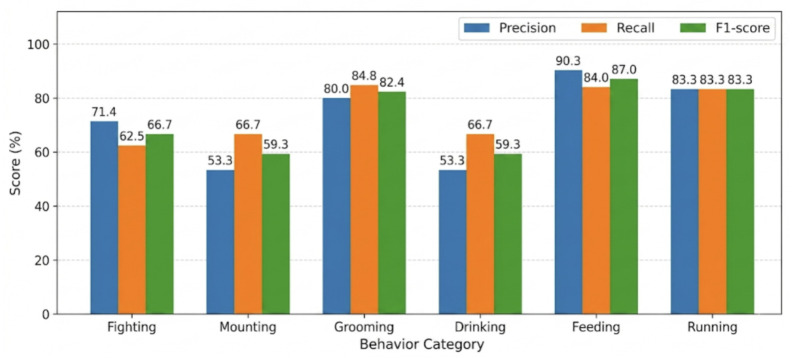
The per-class precision, recall, and F1-score of STED-Net on the Dark Beef Cattle Actions.

The Precision, Recall, and F1-score of STED-Net for the six beef cattle behaviors are illustrated in Figure 8. Overall, the proposed method achieved a macro-average Precision of 71.96%, a macro-average Recall of 74.67%, and a macro-average F1-score of 72.99%, indicating that the proposed method achieved relatively stable recognition performance across different beef cattle behaviors. Specifically, feeding, grooming, and running achieve relatively better overall performance, with F1-scores of 87.0%, 82.4%, and 83.3%, respectively. In contrast, The relatively poor performance is mainly observed for mounting, drinking, and fighting. mounting and Drinking obtain the lowest F1-scores, both at 59.3%, mainly because their local postures are easily affected by occlusion, low contrast, and target overlap under nighttime conditions. In particular, mounting often involves short-duration body contact, while drinking is characterized by a head-lowering posture near fixed facilities, making them difficult to distinguish from other behaviors. Fighting achieves a slightly higher F1-score of 66.7%, but its recall remains limited, which may be related to rapid movements, motion blur, and partially occluded interaction regions in low-light scenes.

**Figure 9:**
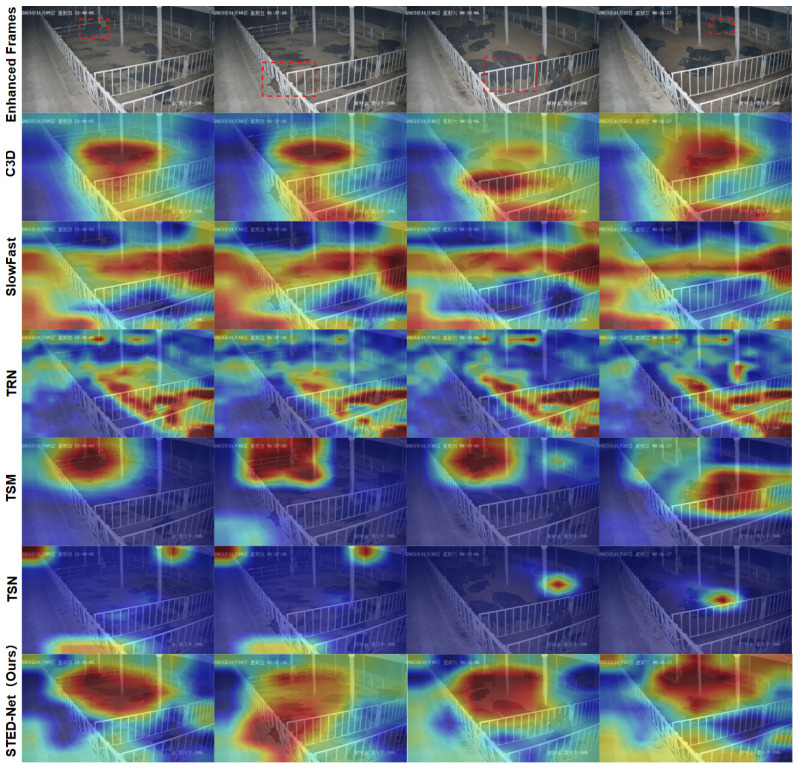
The classification activation mapping for different behavior recognition models. The first row shows representative key frames, in which the behavior regions are marked with red dashed boxes for better visualization. The second to seventh rows present the heatmaps generated by different models. In the heatmaps, red regions indicate the areas receiving the highest attention, whereas yellow regions represent areas with relatively lower but still significant attention.

To more intuitively demonstrate the effectiveness of the STED-Net, Figure 9 presents heatmap visualizations of the aforementioned comparison methods on the test set. The first row shows key frames extracted from different video sequences, while the second to seventh rows correspond to the heatmaps generated by different models. In the heatmaps, red regions indicate areas with the highest attention, whereas yellow regions represent areas with relatively lower but still significant attention. The comparison results show that the STED-Net is able to focus more accurately on the regions where behaviors occur, whereas other methods struggle to effectively attend to the active behavioral regions of the cattle. These heatmap visualizations further confirm that the STED-Net exhibits superior recognition capability.

### 3.2. Visualization and Analysis of Results

**Figure 10:**
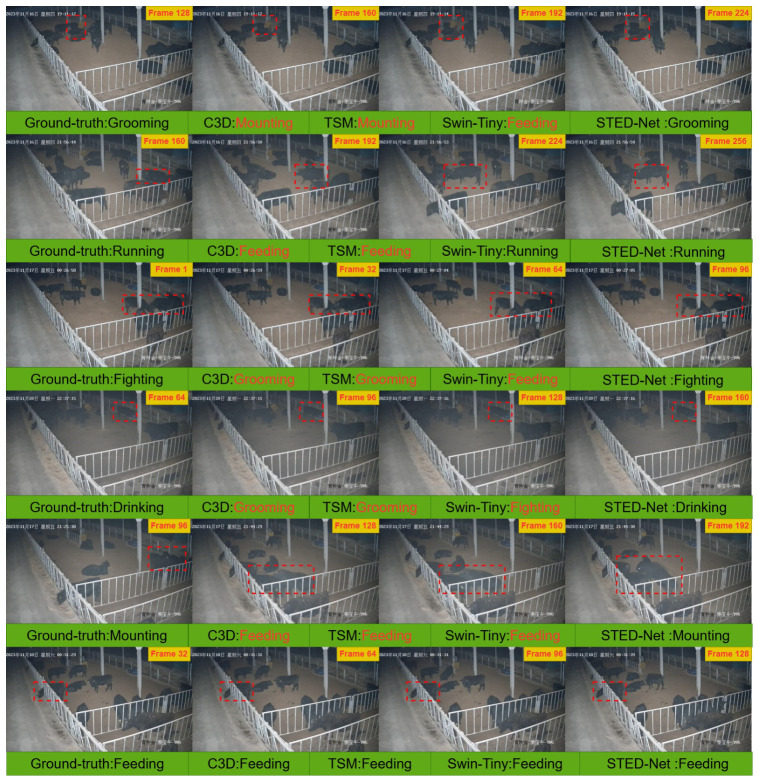
Visualization recognition results of beef cattle behavior. Each row shows four key frames selected from a single video clip, arranged in temporal order to illustrate the dynamic changes of the corresponding cattle behavior. For convenient understanding, the behavior area is marked with a red dashed box.

To intuitively demonstrate the effectiveness of the proposed method, Figure 10 presents qualitative comparison results of cattle behavior recognition on several representative video sequences. Here, the proposed STED-Net is compared with several mainstream behavior recognition models, including C3D [35], TSM [40], and Swin-Tiny [43], where incorrectly predicted class labels are highlighted in red.

Specifically, in the second row of Figure 10, the ground-truth label of the second video sequence is running. It can be observed that C3D and TSM fail to correctly classify this behavior, whereas STED-Net and Swin-Tiny achieve accurate predictions. This performance gap mainly stems from the limited capability of C3D and TSM to model complex motion patterns in the spatio-temporal domain, which makes it difficult for them to fully capture the continuous and prominent dynamic characteristics of running behavior. Further analysis of the first and fourth rows in Figure 10 indicates that STED-Net, by incorporating an image enhancement mechanism, effectively improves the representation of behavioral features in regions that are distant from the camera and characterized by low contrast and insufficient illumination, thereby enhancing its robustness in complex visual environments. For the feeding behavior shown in the last row of Figure 10, all methods are able to correctly recognize the behavior due to the sufficient number of training samples and the relatively high overall brightness of the scene. Overall, these qualitative results demonstrate that STED-Net exhibits superior discriminative capability and higher recognition accuracy compared with other competing methods in beef cattle behavior recognition tasks.

**Figure 11:**
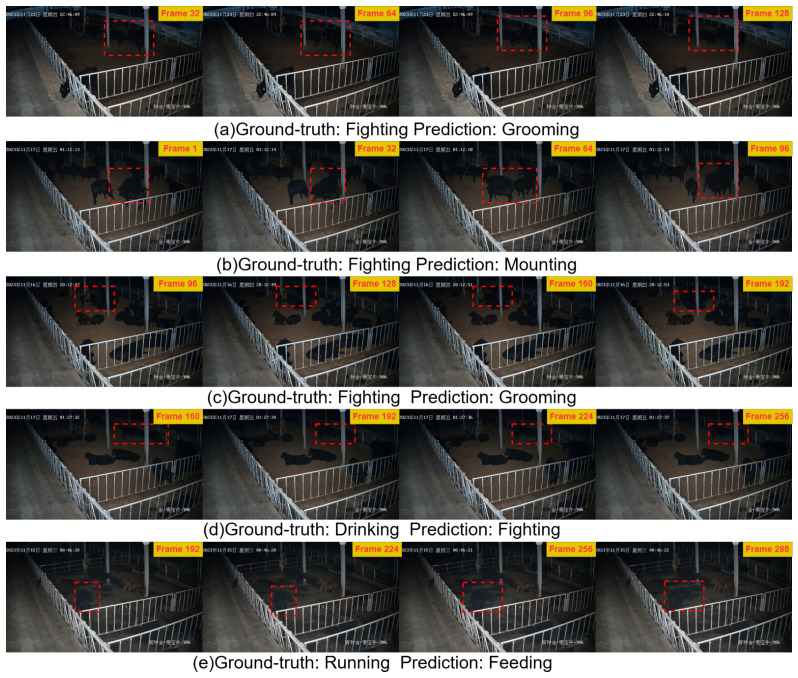
Failure cases of the STED-Net method. Each row shows four key frames selected from a single video clip, arranged in temporal order to illustrate the dynamic changes of the corresponding cattle behavior. For convenient understanding, the behavior area is marked using a red dashed box.

Figure 11 illustrates several representative failure cases of the proposed STED-Net method on cattle behavior recognition. As shown in Figure 11a, STED-Net incorrectly classifies a video with the ground-truth label of grooming as fighting behavior. This misclassification is mainly attributed to the presence of other cattle within the spatial region where the grooming behavior occurs, while the corresponding image region is extremely dark. In addition, due to the camera viewpoint, partial overlap between cattle heads is observed, which causes the grooming behavior to be incorrectly recognized as fighting. As shown in Figure 11b, STED-Net misidentifies a video labeled as fighting as mounting behavior. One possible reason is that fighting behavior often involves interactions among multiple cattle individuals. This makes the behavioral patterns more complex. Meanwhile, occlusion is likely to occur during the interaction. As a result, the extraction and discrimination of motion features become more difficult. As shown in Figure 11c, the fighting behavior is incorrectly classified as grooming. This may be caused by the long camera distance, weak illumination, and partial occlusion in the interaction region. Under these conditions, the head-contact and pushing actions between cattle become less distinguishable, making them visually similar to grooming-related movements. As shown in Figure 11d, the drinking behavior is incorrectly classified as fighting. Under low-light conditions and complex background interference, the head-lowering posture during drinking exhibits certain similarities to head-contact interactions. In addition, occlusion caused by surrounding cattle and farm facilities further increases the difficulty of distinguishing between these two behaviors. As shown in Figure 11e, the running behavior is incorrectly recognized as feeding. This may be because the target cattle are partially occluded by the fence and located in a low-light region. In addition, the short visible motion trajectory and blurred body appearance weaken the motion cues of running, causing the model to confuse it with feeding behavior near the pen area.

### 3.3. Ablation Study

#### 3.3.1. Effectiveness of the STE-Module and DF-Block

The proposed method consists of two key components: the Spatio-Temporal Enhancement Module (STE-Module) and the Dynamic Fusion Block (DF-Block). Table 3 presents the ablation results of these two key components on the test set. Here, the DarkLight framework [44] was adopted as the baseline algorithm, which did not enable the STE-module and DF-Block. The DarkLight framework was selected as the baseline because it is a representative method for action recognition under dark and low-light video conditions, which is consistent with the nighttime beef cattle behavior recognition scenario investigated in this study. Although it was originally proposed for human action recognition, it shares similar video-based spatio-temporal recognition challenges with beef cattle behavior recognition. Therefore, it was adopted as a relevant baseline in this study.

As shown in Table 3, after introducing the DF-Block, the model improves the F1-score by approximately 2.50% (**#**2 vs. **#**1). Moreover, our STE-Module contributes total improvements of 3.94%, 3.63%, 1.59%, and 3.79% in Precision, Recall, Accuracy, and F1-score, respectively (**#**3 vs. **#**1). When further adding STE-Module based on **#**2, it achieves a 7.23% absolute increase in the Accuracy (**#**4 vs. **#**2). Overall, both the DF-Block and the STE-Module contribute positively to the beef cattle behavior recognition in the dark. The full model incorporating all components (**#**4) achieves the best overall performance among all compared settings.

**Figure 12:**
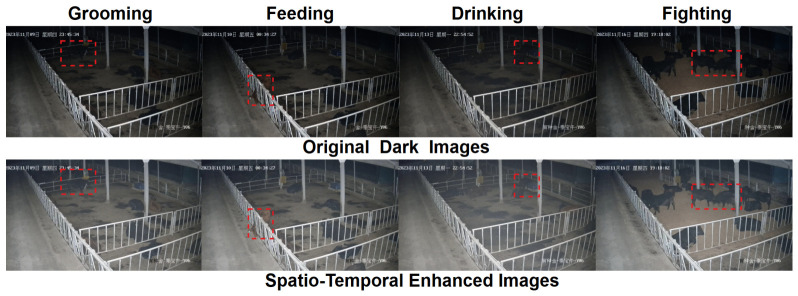
Visual comparison of the original dark image and the spatio-temporally enhanced image. After spatio-temporal enhancement, the details in the dark areas are significantly brightened. For convenient understanding, the behavior area is marked with a red dashed box.

To further verify the effectiveness of the proposed STE-Module, Figure 12 presents its enhancement results. As shown in Figure 12, after processing by the STE-Module, the details in dark regions become clearer and the overall visibility is improved. This result further explains the performance gain brought by the enhanced branch.

#### 3.3.2. Effectiveness of Different Branches in Dynamic Fusion Block

Table 4 presents the performance of the model under different input branches configurations in the dynamic fusion block on the test set. Overall, the results indicate that the three branches contain complementary information, and the model performance is progressively improved as more informative input representations are incorporated. Under the single-branch setting, the enhanced branches achieve higher Recall and F1-score than the original low-light branch, indicating that enhancement helps improve behavioral feature representation under low-light conditions. Under the dual-branch setting, the combination of the Dark-branch and STE-branch achieves the best performance. It is noted that the HE-only branch obtains a higher F1-score than the STE-only branch. This result is reasonable because the HE branch provides a basic and stable global spatial contrast enhancement, which helps improve the visibility of dark frames. However, HE is essentially a spatial enhancement operation and does not explicitly model temporal variations or inter-frame motion information. In contrast, the STE branch is designed to capture spatio-temporal dynamics and temporal correlations under low-light conditions. Although the STE-only branch may be limited when used independently due to insufficient spatial contrast enhancement, its advantage lies in modeling motion-related and temporal contextual features. Furthermore, when all three branches are jointly used, the model obtains the best overall results, with Precision, Recall, Accuracy, and F1-score reaching 88.47%, 80.18%, 83.80%, and 84.12%, respectively. This demonstrates that the original low-light branch, the STE-enhanced branch, and the HE-enhanced branch provide complementary information for behavior recognition in complex low-light environments.

#### 3.3.3. Effectiveness of the Dynamic Fusion Block

Table 5 presents the ablation results under different feature fusion strategies. Overall, the results show that the choice of fusion strategy has a clear impact on model performance. Compared with direct concatenation (Concat), the introduction of Cross-Attention and AFF [31] leads to consistent improvements across all evaluation metrics, indicating that more effective feature interaction and fusion mechanisms are beneficial for behavior recognition. By contrast, when the DF-Block is adopted, the model achieves the best results in terms of Precision, Recall, Accuracy, and F1-score, reaching 88.47%, 80.18%, 83.80%, and 84.12%, respectively. These results demonstrate that the proposed DF-Block can more effectively integrate the complementary information among multi-branch features and thereby improve behavior recognition performance in complex low-light environments.

### 3.4. Robustness and Generalization Analysis

#### 3.4.1. Robustness Analysis Under Different Weather Conditions

Inspired by existing studies [45,46,47] on weather-related image degradation and model robustness evaluation, this study generated three simulated adverse-weather test sets based on the original test videos, including foggy, dusty, and rainy conditions. Specifically, weather degradation operations were applied to the original video frames to simulate visual degradation effects such as fog occlusion, dust coverage, and rain streak interference. As a result, the corresponding foggy, dusty, and rainy test sets were obtained. During the generation process, the behavior category labels of the original videos were kept unchanged to ensure semantic consistency across different weather conditions.

**Figure 13:**
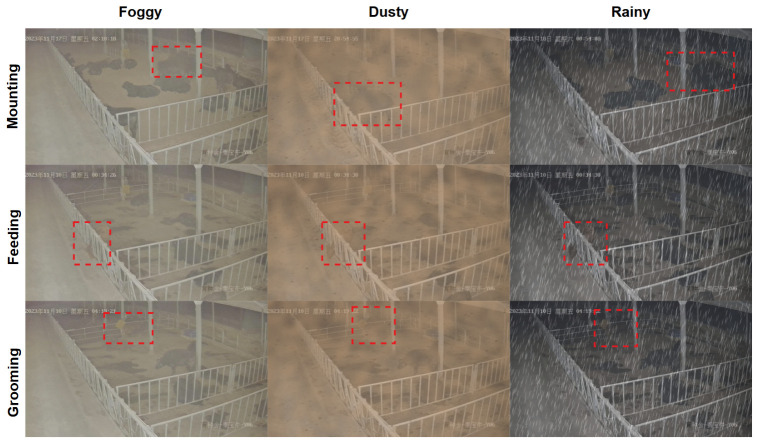
Examples of cattle behavior samples under complex weather conditions. The columns represent foggy, dusty, and rainy conditions, while the rows represent mounting, feeding, and grooming behaviors. The red dashed boxes indicate the main regions where cattle behaviors occur.

Figure 13 shows representative cattle behavior samples under foggy, dusty, and rainy conditions. It can be observed that foggy and dusty conditions reduce the visual difference between cattle targets and the background, making the behavior regions more ambiguous. In rainy scenes, rain streak occlusion further damages local texture information and target edge structures in video frames. These visual degradation effects increase the difficulty of cattle behavior recognition.

In the experimental setting, the generated adverse-weather data were not used for retraining the model. Instead, the trained model was directly applied to the three weather-degraded test sets for inference. Table 6 presents the robustness analysis of the proposed method under different simulated adverse weather conditions, including foggy, dusty, and rainy scenarios. Overall, the model shows different levels of performance degradation under different weather perturbations, indicating that environmental degradation has a clear influence on cattle behavior recognition.

Among the three weather conditions, the model achieves the best performance under foggy conditions, with Precision, Recall, Accuracy, and F1-score reaching 84.21%, 76.35%, 79.58%, and 80.09%, respectively. This result suggests that the proposed method can still maintain relatively stable recognition performance when the visual degradation mainly appears as reduced contrast and blurred target boundaries. Under rainy conditions, the model obtains a Precision of 77.80%, a Recall of 69.50%, an Accuracy of 72.50%, and an F1-score of 73.40%. Although the rain streaks introduce local occlusion and interfere with target edge information, the model still retains a certain recognition ability. In contrast, the performance under dusty conditions decreases more significantly, with the F1-score dropping to 52.10%. This indicates that dust interference causes more severe visual degradation than fog and rain in this experiment. The dust coverage changes the global color distribution of the video frames, weakens the contrast between cattle targets and the background, and obscures local behavioral details. These factors make it more difficult for the model to extract reliable spatio-temporal features. Overall, the results demonstrate that the proposed method has a certain degree of robustness under complex weather perturbations, but severe dust interference remains a challenging condition for nighttime cattle behavior recognition.

#### 3.4.2. Cross-Farm Generalization Analysis

To further evaluate the adaptability of the proposed method under different data sources, this study selected the nighttime subset of Beef Cattle Abnormal Actions [24] as external dataset. Further training and testing were then conducted on this subset. The nighttime subset differs from the constructed Dark Beef Cattle Actions in terms of collection scenario, camera viewpoint, background layout, target scale, and imaging quality.

**Figure 14:**
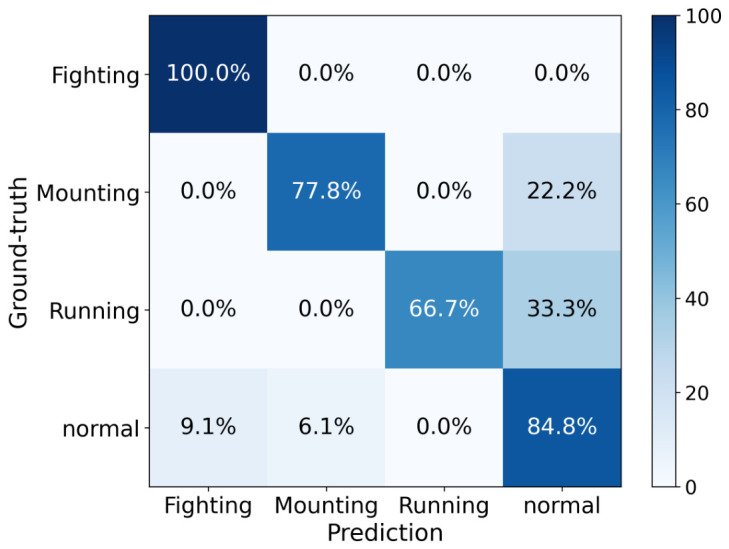
Confusion matrix of behavior recognition using the STED-Net on the Nighttime Subset of Beef Cattle Abnormal actions [24].

The confusion matrix of the proposed STED-Net method on the Nighttime Subset of Beef Cattle Abnormal actions is illustrated in Figure 14, The Figure 14 shows that the proposed method maintains a reasonable recognition performance on the external dataset. Fighting achieves the best classification result, with all samples correctly recognized. The Normal category also obtains a relatively high recognition accuracy of 84.8%, although a small proportion of samples are misclassified as Fighting and Mounting. This may be due to occasional body contact, posture overlap, or local interactions among cattle during normal activities. In comparison, Mounting and Running show relatively lower recognition performance, with 22.2% and 33.3% of samples misclassified as Normal, respectively. This suggests that short-duration behaviors or behaviors with weak motion cues are more likely to be missed under cross-farm conditions. Overall, the results indicate that the proposed method has a certain generalization ability in external nighttime scenarios, but its recognition of transient and motion-sensitive behaviors remains limited when the visual appearance, camera perspective, or farm environment changes.

**Figure 15:**
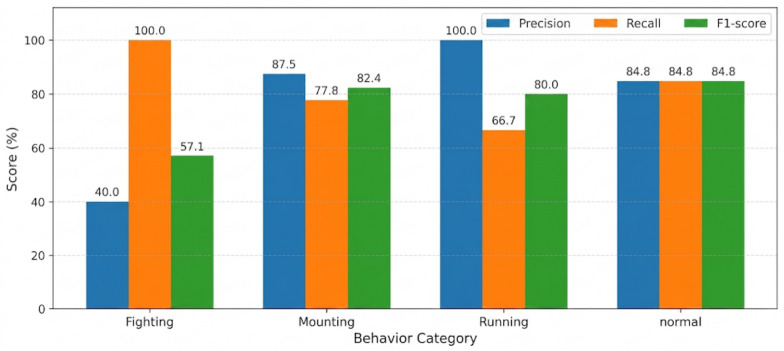
The per-class precision, recall, and F1-score of STED-Net on the Nighttime Subset of Beef Cattle Abnormal actions [24].

Following the same training strategy, input configuration, and experimental settings used in the preceding experiments, the proposed method achieved an Accuracy of 82.14%, a macro-averaged Precision of 78.09%, a macro-averaged Recall of 82.32%, and a macro-averaged F1-score of 76.09% on this subset. These results indicate that the proposed method can maintain a certain level of recognition performance when applied to nighttime data from a different source. The class-wise results are further shown in Figure 15. The Normal category achieved 84.8% in Precision, Recall, and F1-score, indicating relatively balanced recognition performance. For the Mounting category, the Precision, Recall, and F1-score were 87.5%, 77.8%, and 82.4%, respectively. The Running category obtained a Precision of 100.0%. However, its Recall was 66.7%, suggesting that some true Running samples were misclassified as other categories. In contrast, the Fighting category achieved a Recall of 100.0%, but its Precision was only 40.0%, with an F1-score of 57.1%. Overall, the proposed method showed relatively stable performance on the Mounting, Running, and Normal categories, whereas the recognition of Fighting behavior remained less reliable. This may be related to the limited number of Fighting samples, the large variation in motion patterns, and the visual similarity between Fighting and other intense movement behaviors.

**Figure 16:**
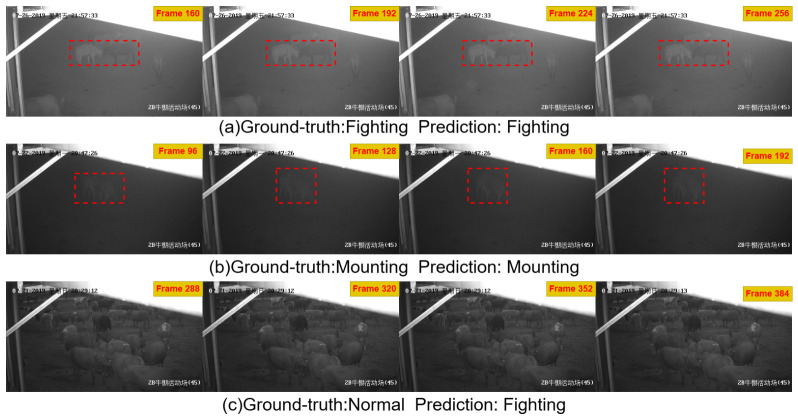
Examples of cattle behavior recognition results on the Nighttime Subset of Beef Cattle Abnormal Actions [24]. For convenient understanding, the behavior area is marked with a red dashed box.

Figure 16 presents several representative recognition results of the proposed method on the subset of Beef Cattle Abnormal Actions [24], which further illustrates its performance under different data sources. Specifically, Figure 16a,b show correctly classified samples of Fighting and Mounting, respectively. It can be observed that although the external nighttime samples commonly suffer from low illumination, small behavioral regions, and blurred behavior boundaries, the proposed method correctly classifies these samples. This indicates that the proposed method has certain behavior representation ability and data adaptability in external nighttime scenarios. Figure 16c shows a failure case in which a Normal sample is misclassified as Fighting. In this scene, multiple cattle are present, with occlusion, overlap, and close physical contact among individuals. Some normal group activities show local visual patterns similar to Fighting behavior. Meanwhile, the low illumination at night reduces image details and further weakens the difference between normal contact and abnormal aggressive interaction, leading to misclassification. This result suggests that the proposed method performs well in recognizing typical abnormal behaviors, but it still has certain limitations in crowded external nighttime scenarios with multi-object interactions and ambiguous class boundaries.

## 4. Conclusions

Monitoring and recognizing beef cattle behaviors are of great significance for improving animal health, welfare, and production performance. However, achieving reliable behavior recognition in dark environments remains a challenging task. In this study, we constructed a dataset containing real nighttime beef cattle behaviors, named Dark Beef Cattle Actions. Based on this dataset, the proposed STED-Net method was developed to achieve better recognition performance on the Dark Beef Cattle Actions. The proposed method achieved a Precision of 88.47%, a Recall of 80.18%, an Accuracy of 83.80%, and an F1-score of 84.12%. In addition, comparative experiments with several recent state-of-the-art behavior recognition methods, as well as additional robustness and generalization experiments, further demonstrated the effectiveness of the proposed STED-Net framework. In future work, more beef cattle behavior data from both indoor and outdoor environments will be collected to alleviate the problem of sample imbalance. In addition, class-balancing strategies suitable for the joint enhancement-recognition training framework will be further investigated to improve the recognition performance of minority behaviors without affecting the stability of the enhancement module. Moreover, although the proposed method achieves promising recognition performance, its parameter efficiency and inference speed can be further improved. Therefore, future work will investigate more lightweight architectures to enhance computational efficiency while preserving recognition accuracy.

## Figures and Tables

**Table 1:** Criteria for judging behavior of Dark Beef Cattle Action datasets.

Behavior Category	Behavioral Definition
Running	Beef cattle perform rapid and continuous spatial movement at a speed clearly higher than normal walking.
Feeding	Beef cattle approach the feed trough and extend their heads into the trough area for feed intake.
Drinking	Beef cattle approach the water trough and extend their heads into the trough area for water intake.
Grooming	Beef cattle approach the grooming brush and rub their body against the brush repeatedly.
Mounting	A beef cattle raises the front leg and mounts another beef cattle.
Fighting	Beef cattle show aggressive interactions with other individuals, such as head-butting, pushing, chasing, or physical confrontation.

**Table 2:** Comparison with other state-of-the-art behavior recognition methods based on the Dark Beef Cattle Actions dataset. The best results are highlighted in bold.

Method	Precision	Recall	Accuracy	F1-Score	Parameters (M)	Inference Time (ms)
C3D	85.09%	70.14%	66.83%	76.89%	78.02	40.92
TSN	66.99%	47.51%	39.48%	55.59%	**23.52**	**19.63**
I3D	67.98%	56.65%	45.38%	61.80%	35.40	56.69
R(2 + 1)D	62.26%	33.64%	38.58%	43.68%	63.76	127.46
TRN	75.00%	57.01%	44.14%	64.78%	26.64	60.61
SlowFast	69.07%	58.47%	47.57%	63.33%	42.10	105.61
TSM	86.75%	69.23%	65.96%	77.01%	23.86	32.10
TAM	69.35%	56.54%	48.48%	62.29%	25.59	282.46
TPN	70.38%	57.92%	46.87%	63.54%	91.50	329.27
Swin-T	73.30%	58.73%	74.50%	65.21%	88.83	123.20
STED-Net (ours)	**88.47%**	**80.18%**	**83.80%**	**84.12%**	178.73	292.26

**Table 3:** Experimental results of the effectiveness of the proposed module. The checkmark symbol indicates that the corresponding module is activated in the ablation setting. The best results are highlighted in bold.

#	Baseline	DF-Block	STE-Module	Precision	Recall	Accuracy	F1-Score
1	✓			81.82%	71.82%	76.59%	76.49%
2	✓	✓		83.87%	74.65%	76.57%	78.99%
3	✓		✓	85.76%	75.45%	78.18%	80.28%
4	✓	✓	✓	**88.47%**	**80.18%**	**83.80%**	**84.12%**

**Table 4:** Experimental results of different enhancement branches and combinations, where Dark-branch denotes the original low-light input, STE-branch represents the STE-enhanced input, and HE-branch corresponds to the histogram equalization–enhanced input. The checkmark symbol indicates that the corresponding branch is activated in the ablation setting. The best results are highlighted in bold.

#	Dark-Branch	STE-Branch	HE-Branch	Precision	Recall	Accuracy	F1-Score
1	✓			83.61%	47.51%	78.35%	60.59%
2		✓		76.09%	72.73%	74.09%	74.37%
3			✓	84.39%	75.00%	70.02%	79.42%
4	✓	✓		85.99%	75.00%	80.77%	80.12%
5	✓		✓	79.17%	65.46%	65.98%	71.66%
6		✓	✓	83.86%	68.64%	75.70%	75.49%
7	✓	✓	✓	**88.47%**	**80.18%**	**83.80%**	**84.12%**

**Table 5:** Ablation study with different feature fusion strategies. The best results are highlighted in bold.

Feature Fusion Function	Precision	Recall	Accuracy	F1-Score
STED-Net with Concat	79.61%	73.64%	76.82%	76.51%
STED-Net with Cross-Attention	81.45%	75.46%	79.43%	78.34%
STED-Net with AFF	82.25%	76.23%	80.83%	79.13%
STED-Net with DF-Block (ours)	**88.47%**	**80.18%**	**83.80%**	**84.12%**

**Table 6:** Robustness analysis under simulated adverse weather conditions.

Weather Conditions	Precision	Recall	Accuracy	F1-Score
Foggy	84.21%	76.35%	79.58%	80.09%
Dusty	58.10%	47.30%	50.20%	52.10%
Rainy	77.80%	69.50%	72.50%	73.40%

## Data Availability

The data presented in this study are available upon request from the corresponding author. The data are not publicly available due to the privacy policy of the authors’ institution.
